# Spatiotemporal variation in the fecal microbiota of mule deer is associated with proximate and future measures of host health

**DOI:** 10.1186/s12917-021-02972-0

**Published:** 2021-07-29

**Authors:** Hyrum S. Eddington, Courtney Carroll, Randy T. Larsen, Brock R. McMillan, John M. Chaston

**Affiliations:** Department of Plant and Wildlife Sciences, Brigham Young University, Provo, UT USA

**Keywords:** Ruminant, Fat storage, Body condition

## Abstract

**Background:**

Mule deer rely on fat and protein stored prior to the winter season as an energy source during the winter months when other food sources are sparse. Since associated microorganisms (‘microbiota’) play a significant role in nutrient metabolism of their hosts, we predicted that variation in the microbiota might be associated with nutrient storage and overwintering in mule deer populations. To test this hypothesis we performed a 16S rRNA marker gene survey of fecal samples from two deer populations in the western United States before and after onset of winter.

**Results:**

PERMANOVA analysis revealed the deer microbiota varied interactively with geography and season. Further, using metadata collected at the time of sampling, we were able to identify different fecal bacterial taxa that could potentially act as bioindicators of mule deer health outcomes. First, we identified the abundance of *Collinsella* (family: *Coriobacteriaceae*) reads as a possible predictor of poor overwintering outcomes for deer herds in multiple locations. Second, we showed that reads assigned to the *Bacteroides* and *Mollicutes* Order RF39 were both positively correlated with deer protein levels, leading to the idea that these sequences might be useful in predicting mule deer protein storage.

**Conclusions:**

These analyses confirm that variation in the microbiota is associated with season-dependent health outcomes in mule deer, which may have useful implications for herd management strategies.

**Supplementary Information:**

The online version contains supplementary material available at 10.1186/s12917-021-02972-0.

## Background

Mule Deer (*Odocoileus hemionus*) are indigenous to the western United States and Canada. Populations have experienced significant fluctuations over the past one-half century and there is evidence of an overall decline in numbers [1]. Many factors contribute to fluctuations in herd size including maternal investment in reproduction, predation, disease, accidents (i.e.., road kill), winter severity, and others [2–6]. However, it is likely that the primary driver of population dynamics is herd health or the availability of sufficient food for growth, maintenance, and reproduction [7]. In fact, condition (e.g., ingesta free body fat (IFBF) of female mule deer is linked to life-history characteristics including over-winter survival, birthweight of offspring, and survival of offspring [8–11]. Understanding the effects of management decisions on health or condition of mule deer are major initiatives of governmental agencies and non-governmental organizations throughout the range of this species.

Fat acquisition and storage is a crucial aspect of individual survival for mule deer and other ungulates. With lower access to energy and nutrients during the winter months, deer first utilize fat stores and then protein mass for energy during winter. For example, fat levels may increase from between 9 to 25% from June to December, with a then steady decrease from January to April [12]. This pattern of fat deposition in deer is well-established and tied to the scarcity of forage during winter months. In addition to food scarcity, seasonal changes in mule deer fat deposition may also be related to the microorganisms associated with the deer, especially in the gastrointestinal tract (the ‘microbiota’). The gut microbiota of ruminants, including deer, can directly impact the health of an individual deer and variation in the identity and abundance of specific microoorganisms can be affected by differences in host genotype, geography, age, gender, and diet ([13–18]). Ruminants depend on their gut microbiota for up to 70% of the energy extracted from their diet [19] and 95% of the rumen microbiome is made up of bacteria, mostly anaerobes or facultative anaerobes [20]. Two of the most common bacterial phyla in ruminant guts are involved in digestion: the Firmicutes break down fibers such as cellulose and the Bacteroidetes digest proteins and polysaccharides [21]. Ruminants lack several enzymes necessary for digestion and it is believed that gut symbionts perform these functions. Thus, the microbiota is essential for deer health and metabolism. Currently there are no known microbial markers that directly link the gut microbiota and mule deer overwintering success.

Our purpose was to test if the deer gastrointestinal microbiota varies with season (winter vs. spring) and to identify specific microorganisms, if any, that are related to individual deer survival. We hypothesized that the microbiota would vary significantly with season and expected that specific microorganisms that varied in abundance, if detected, could be potential bioindicators of mule deer health. As part of the approach we asked two specific questions about the microbiota of two Utah mule deer herds: 1) Does the microbiota of individual deer vary with geography and season? and 2) Can variation in the microbiota predict proximate or future measures of deer health?

## Results

### The microbiota of two Utah mule deer populations varies with geography and season

**Fig. 1:**
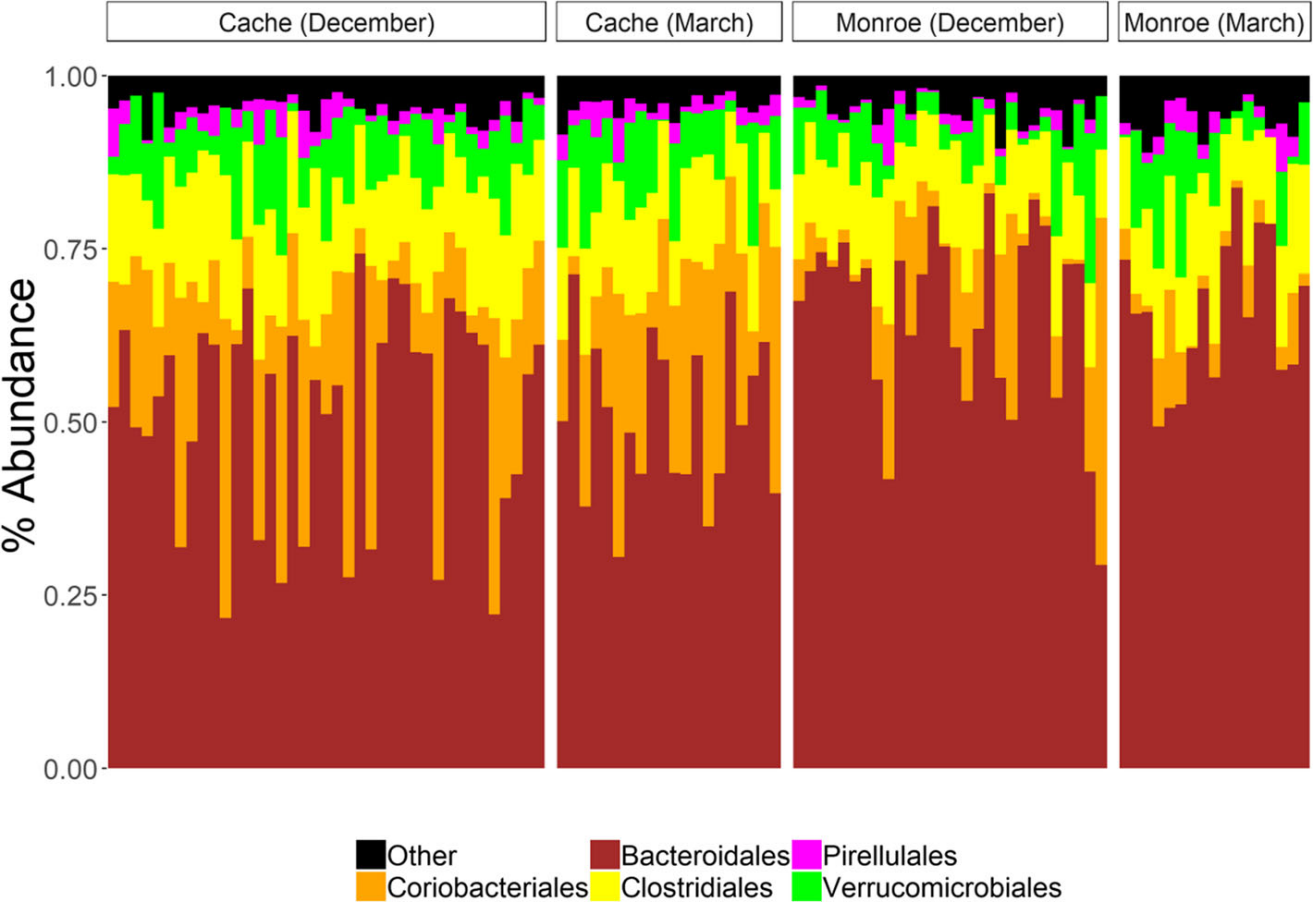
Microbial communities of the Cache and Monroe Valley mule deer populations. Taxonomic composition of the fecal samples, shown at the order level by geographic location and sampling date

**Fig. 2:**
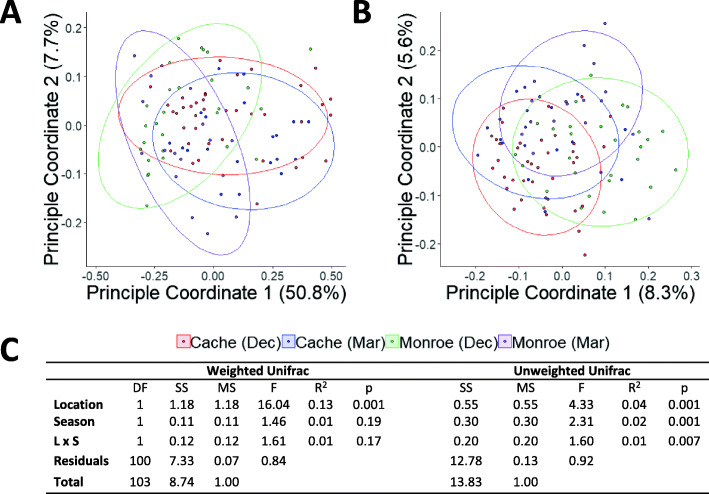
Location- and season-dependent variation in Cache and Monroe Valley mule deer populations. Principal coordinate analysis of samples from Cache and Monroe valley for **A**) weighted and **B**) unweighted Unifrac distances. **C**) PERMANOVA statistics corresponding to each plot are also shown (degrees of freedom (DF), sum of squares (SS), mean sum of squares (MS), F-value (F), *p*-value (P), location by season interaction (L x S))

To better understand how location and season contribute to differences in the deer microbiota, we performed a 16S rRNA gene survey of 108 deer samples collected from herds located in Monroe (*n* = 48) and Cache (*n* = 60) valley (location) during December and March (season) (Table 1). A total of 3,404,323 bacterial reads and 4266 amplicon sequence variants (ASVs) were obtained on a partial Illumina HiSeq lane, with a median of 24,621 reads per sample. The most obvious differences in the microbiota were the increased abundance of *Coriobacteriales* in the Cache herd versus the Monroe herd (Fig. 1, Supplementary Tables 1–2). To identify factors associated with differences in the overall microbiota composition the microbiota of the different populations was compared using principal coordinate analysis (PCoA) of Unifrac distances in 13,000-read rarefied samples (Fig. 2A-B), which was near-saturating sampling (Figure S1). Microbiota composition varied significantly with both geography and season by identity of the associated microbes, and only by geography when microbial abundance was considered (Fig. 2, Supplementary Table 3). The significant interaction term in the unweighted Unifrac distance analysis suggests that the microbiota responses to seasonal variation were geography specific (*p* < 0.05). Together, these data showed a significant effect of both geography and season on the microbiota of mule deer.

**Table 1:** Table of Sub-Samples and Core ASVs (90% of samples)

Site	Month	# of individuals	Average IFBF score	Total Core ASVs	Taxonomic assignments of core ASVs
Cache	Dec 15	40	11.0	10	Unassigned genera 5-7 N15, A*dlercreutzia, Anaerofilum, Anaerotruncus, Coriobacteriaceae, Mogibacterium, Phascolarctobacterium* Unassigned families Mogibacteriaceae, Rikenallaceae, Ruminococcaceae
Cache	Mar 16	20	6.3	12	Unassigned genera 5-7 N15, *Adlercreutzia, Anaerofilum, Anaerotruncus, Mogibacterium, Phascolarctobacterium,* Unassigned families Coriobacteriaceae, Mogibacteriaceae, Pirellulaceae, Rikenallaceae,Ruminococcaceae Unassigned order Clostridiales
Monroe	Dec 15	29	9.0	4	Unassigned genera 5-7 N15, *Phascolarctobacterium,* Unassigned familes Mogibacteriaceae, Rikenallaceae
Monroe	Mar 16	19	5.8	4	Unassigned genera g_5-7 N15, *Anaerofilum, Anaerotruncus,* Unassigned family Mogibacteriaceae

We defined the core microbiota in different groups of sampled animals. Some bacterial phyla were present in every sample, including Bacteroidetes, Firmicutes, Actinobacteria, Verrucomicrobia, Cyanobacteria, and Proteobacteria. No ASVs were detected in every individual, but 4–12 core ASVs were detected in 90% of the animals in the different pools of animals grouped by season and geography (Table 1). In particular, samples from Cache Valley in both December and March showed multiple Coriobacteriaceae ASVs that were not present in the core microbiota of the Monroe Valley deer. As a whole, a search for a core microbiota revealed uniformity at the phylum level and ASV-level distinctions based on geographic location and season.

### Coriobacteriaceae are associated with post-winter fat levels

**Fig. 3:**
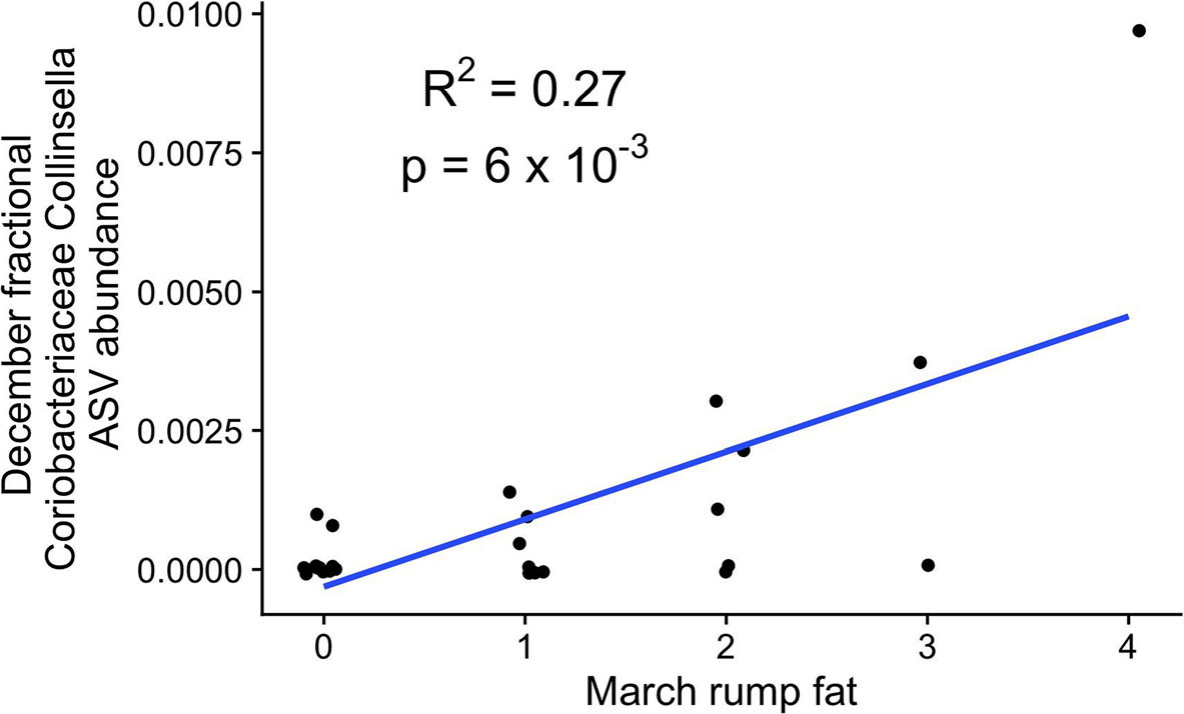
Coriobacteraceae abundance predicts future mule deer rump fat. Dotplot shows the relationship between Coriobacteriaceae abundance in deer sampled in December, and rump fat of deer sampled in March. For display, points were jittered the around the x-axis by 0.1 units and around the y-axis by 8 × 10^− 5^ units

To test if certain microbes might predict mule deer health, we calculated the relationship between the relative abundances of ASVs detected in deer in December and three health and nutritional measures in the same deer approximately 3 months later: rump fat, loin thickness, and body condition score (BCS) (Supplementary Tables 4–8). No bacterial ASVs or higher-level designations were significantly associated with variation in any of these three traits. However, when immature deer (< 2 years old) were removed from the analysis, read counts assigned to an unspecified species in the genus Collinsella, family Coriobacteriaceae, (Fig. 3) in December deer were positively associated with rump fat levels of the same individuals 3 months later (Fig. 3, FDR-corrected *p* < 0.05). This finding suggests that this ASV from the family Coriobacteriaceae may be a bioindicator of fat storage and consequently linked to outcomes such as survival and reproduction that are associated with increased levels of deer rump fat.

### Individual ASVs are associated with mule deer protein storage

**Fig. 4:**
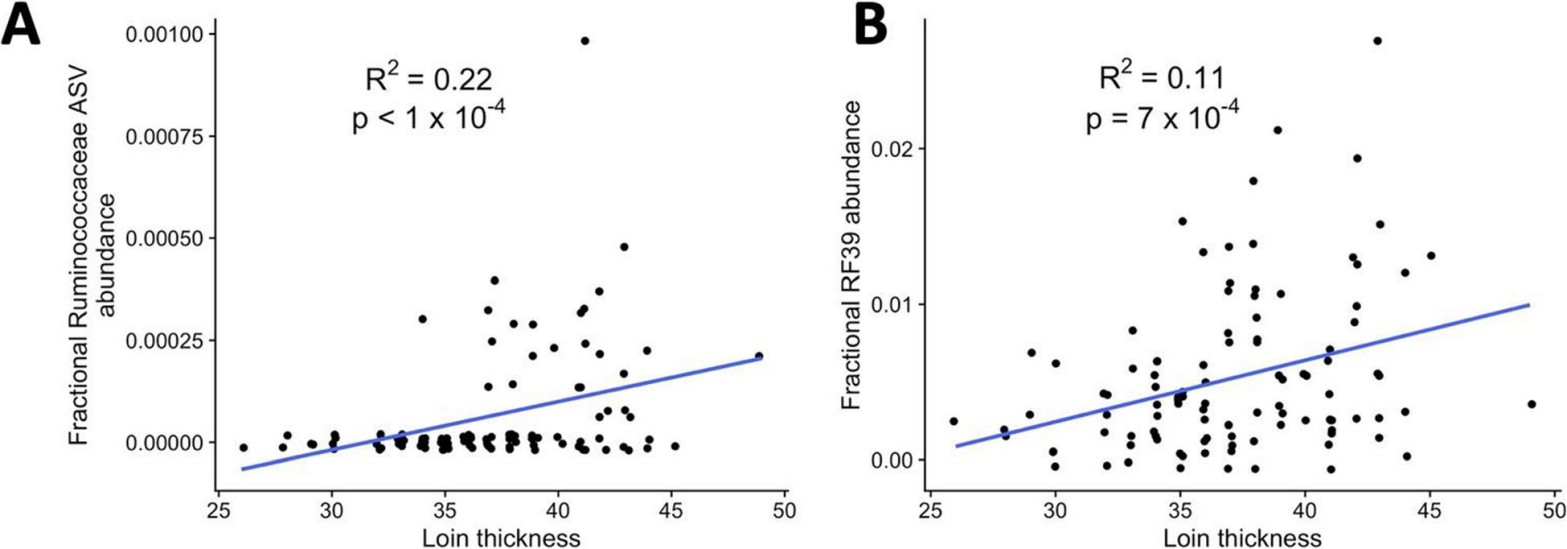
Mule deer loin thickness is correlated with individual ASV abundances. Scatterplots of Loin Thickness with a **A**) Ruminococcaceae ASV and **B**) ASVs clustered at the Order levels as unclassified abundance. All values were obtained from tables rarefied to 13, 000 reads. For display, points were jittered the following units around the x- and y- axes, respectively: A) 0.2 and 2 × 10^− 5^; B) 0.1 and 0.001

In addition to future measure of mule deer condition, we also tested if variation in the microbiota was associated with proximate measures of mule deer nutritional health. Spearman rank tests were used to test for correlations between microbial ASVs and deer metadata collected at the same time as the fecal samples. The only correlations that were significant after false-discovery rate correction were for mule deer loin thickness (Supplementary Tables 9–13). The lone correlated ASV was of relatively low abundance (88 reads in the full dataset) and could not be assigned past the family level as a member of the Ruminococcaceae (Fig. 4A). This correlation would not be useful to predict loin thickness from ASV content since the maximum value of the trendline was based on fewer than four of 13,000 reads sampled per deer. Therefore, the analysis was extended to ASVs grouped at higher taxonomic levels, based on the expectation that functional redundancy among conphyletic taxa might mask the detection of significant correlations in ASVs. At higher levels, 243 ASVs belonging to the order RF39 (phylum Tenericutes, class Mollicutes) drove significant positive correlations between loin thickness (a surrogate protein measure) at the order and phylum levels (Fig. 4B, see also Supplementary Tables 9–13). Taken together, these findings suggest that total RF39 ASV abundance in freshly collected fecal pellets is correlated with and can be used to predict proximate measures of protein storage in mule deer across multiple geographies and seasons.

## Discussion

Our goals were to survey the gastrointestinal microbiota of wild mule deer populations in Utah and test for possible relationships between the detected microbial communities and measures of mule deer health. A survey of two different populations of deer in Cache County and Monroe Valley, UT, USA revealed differences in the microbiota between these two herds that varied with the sampling time. Key biological differences between the two herds both before and after winter included fat levels, protein levels, and BCS, suggesting a relationship between herd location and health, accompanied by geographic variation in the microbiota. The identitification of microbial taxa whose abundance was correlated with individual post-winter rump fat levels provides an additional link between microbiota composition and herd health. However, as our data are insufficient to establish which factors are causal, we cannot infer if the associated microbes are responsible for or respond to the differences in BCS; or how deer behaviors (feeding) or environment (diet) may contribute to the observation variation in the microbiota.

Sequencing reads assigned to *Collinsella*, a Coriobacteriaceae genus, predicted future rump fat levels in the deer in this study, suggesting this taxon may be associated with condition of wintering animals. Coriobacteriaceae are known lipid metabolizers [22], and one explanation for the association we detected could be that these bacteria grow better in deer that begin the winter with high fat stores. An alternative view is that if Coriobacteriaceae metabolize lipids then their abundance should be negatively, not positively associated with host fat levels. Instead, the correlation may be related to bacterial utilization of other (e.g. dietary) fat resources, and their greater abundance in some animals may reflect that animals’ consumption of a diet elevated in fat. In either scenario, the data suggest that pre-winter sampling for Coriobactericeae abudances from deer droppings might provide useful insights to predicted deer performance after the winter season and aid in herd management plans by predicting populations likely to require overwintering interventions. One limitation of using sampled feces to predict future rump fat levels is that the correlation was not detected when deer of young ages were included in the analysis, and fecal pellets from 1 to 2 year old deer are not readily distinguished from adult fecal pellets. Overall, further research is necessary to fully test the idea that fecal pellet sampling can be used to predict deer overwintering and to define the relationship between Coriobacteriaceae and deer fat-content.

As loin thickness reflects protein content in individual deer [23], its correlation with RF39 and Ruminoccocaeceae abundance suggests bacteria belonging to these taxa may be related to protein storage or utilization. In other ruminants, RF39 abundance has been positively associated with residual feed intake (RFI), which measures the efficiency of an animal’s feed consumption, and gross feed intake (GFE), which reports an animal’s milk output normalized to dry matter intake [24, 25]. Larger RFI values are associated with excessive food intake and high GFE values suggest increased milk output per unit of food. If these bacteria have the same effects in mule deer, then the positive correlation between RF39 and loin thickness could be associated with increased accumulation or decreased utilization of protein storage, such as through excess feed consumption or increased efficiency. Alternatively, these microbes might causally shape the change in mule deer protein content. Regardless of the basis for the effect, our intitial analysis suggests fecal RF39 counts are a readily accessible bioindicator of mule deer loin thickness. Comprehensive support for this idea awaits further replication, where deer that were not used to calculate the regression are tested simultaneously for loin thickness and microbiota content, and matched for their fit to predictions.

An ideal goal is to economize time and finances by incorporating bioindicators of mule deer health or overwintering outcomes into established herd management strategies. Historical strategies for monitoring deer health included checking zyphoid fat levels at deer check stations or from roadkill animals, which provide relatively poor estimates of condition [26]. More recently, a reliable method of measuring body fat has been developed [26], but the new method requires researchers to capture live animals and estimate condition based on body palpation and multiple measures acquired via ultrasonography. Because of the expense to capture life animals and the expertise required to correctly palpate animals and to make ultrasonography measures, it is difficult to implement such methods at a large scale. In this study, we show that sequencing the microbiota in fresh feces from wild-caught deer identifies taxa that predict proximate and future outcomes of deer. A key remaining gap is to test if older, wild-collected droppings can predict deer health outcomes with similar patterns to fresh samples. If fecal samples that are several days old retain predictive signatures (either those identified here, or possibly distinct ones), it points to the success of this approach.

## Conclusions

In summary, this study surveyed the gut microbiota of mule deer in two locations before and after the winter season. The significant variation in mule deer microbiota composition with geography suggests environmental or dietary characters may contribute to differences in the mule deer microbiota. In addition, when the abundances of specific microbes were different between groups or significantly correlated with mule deer health outcomes, these findings identify ASVs that could putatively function as bioindicators of mule deer health. Further studies that clarify the causal basis for the correlative relationship between these taxa and deer health have the potential to provide further insight into the relationship between season, mule deer fat and protein storage, and the microbiota. Such work is important to better understand narrow issues addressed by this work, such as if mule deer feces can predict ot report mule deer health, as well as broader areas including the relationship between the microbiota and their animal hosts.

## Methods

### Sample collection

Capture and handling procedures were reviewed, evaluated, and approved by by the Brigham Young University’s (BYU) Animal Care and Use Committee (protocol 150,110). All capture and handling procedures complied with the approved methods. Samples were obtained at two different times from each of the two herds in Cache and Monroe valley, once in December and once in March, for 108 total samples. Individual deer sampled from the herds were randomly selected, so that some of the deer were captured twice (see the ‘Animal_ID’ column of Supplementary Table 2; i.e. 75 unique animals were captured of which 32 were captured twice). Measurements and samples were only collected for captured female deer. During handling, deer were weighed and age was estimated via tooth wear and eruption pattern [27, 28]. Preliminary results suggest > 80% accuracy within 2 years when aging mule deer in Utah from tooth wear compared to cementum analysis (Hinton, unpublished data). For condition of mule deer, ingesta free body fat (IFBF) was estimated using the BCS method and equations developed by Cook et al. [29]. This method estimates IFBF from measurements of rump fat via ultrasonography (E.I. Medical Imaging portable ultrasound), estimates of age, body mass, and a BCS from 1 to 6 [29]. In addition, loin thickness was estimated as suggested by Cook et al. [29]. Fecal samples were collected directly from each animal with a gloved hand, and samples from each animal were stored in individual plastic bags and a cooler prior to freezing at − 20 °C. Captures took place in remote locations throughout the State of Utah. Nearly all animals were released near the site of capture. The only animals that were euthanized (~ 2%) were injured during capture such that they could not be released back into the wild. Those individuals were euthanized by authorized State of Utah personnel under the supervision of the State of Utah Wildlife Veterinarian. The procedure typically consisted of shooting the animal with a 0.22 long-rifle mushroom bullet such that the bullet would pass through the brain toward the spine.

### Molecular biology and sequencing

For each of the 108 fecal samples, microbial DNA was isolated from 60 to 80 mg of a homogenized fecal pellet using the ZR-96 Fecal DNA Kit™ (Zymo Research, Irvine, CA; the kit includes a homogenization step) according to manufacturer instructions. 16S rRNA marker gene libraries (V4 region) were prepared exactly as described previously [30]. Briefly, the V4 region was amplified using the AccuPrime *Pfx* SuperMix (Invitrogen, Carlsbad, CA) in combination with dual-barcoded primers (barcodes in Supplementary Table 2; custom (not from Illumina) sequencing primers were used, as described in [30]). PCR amplicons were normalized using the SequalPrep Normalization kit (ThermoFisher Scientific, Waltham, MA) according to manufacturer instructions. Sequencing was performed on a partial lane of a HiSeq 2500 at the BYU DNA Sequencing center. Samples plus 10% PhiX control DNA were sequenced using 2x250bp v2 Illumina sequencing kits. Sequences were deposited to the National Center for Biotechnological Information’s Short Read Archive under study number PRJNA743316 at https://www.ncbi.nlm.nih.gov/bioproject/PRJNA743316.

### Sequence analysis

Demultiplexed samples were obtained from a partial lane of a paired-end 2 × 250 Illumina HiSeq 2500 and analyzed using QIIME2 [31]. Reads were imported into a paired-end QIIME2 artefact, cleaned via denoising and chimera removal with the DADA2 plug-in [32], and clustered into ASVs. Taxonomy was assigned using pre-fitted, sklearn-based, Naïve Bayes classifier [33] trained on the GreenGenes reference base [34, 35] and a phylogenetic tree was built using the QIIME2 Fast Tree phylogeny pipeline [36]. ASV tables were filtered to exclude ASVs assigned to Archaea. Samples were rarefied to 13,000 reads (Fig. 1) and unweighted and weighted Unifrac distances were used to test for statistically significant differences between treatments. The complete ASV table and taxonomic assignments are shown in Supplementary Table 1.

### Statistical analysis

Statistical analyses were performed using QIIME2 and R [37]. Beta diversity was calculated using unweighted and weighted Unifrac distance [38] and differences between samples were confirmed by PERMANOVA using the R package vegan [39]. Differences in ASV abundance between samples were performed using ANCOM [40]. Spearman rank correlations and Kruskal-wallis test for categorical variables were calculated in R between the values of traits estimating deer health (Rump fat, loin thickness, BCS) and the absolute abundance of bacterial ASVs in a 13,000-read rarefied ASV table. The cutoff for *p*-value significance was 0.05 or, where applicable, a false discovery rate (FDR)-corrected *p*-value of 0.05.

## Supplementary Information

**Additional file 1: Table S1**. Rarefied (13,000) OTU’s with counts per sample. **Table S2**. Primer Barcodes and Relevant Metadata for All Samples. **Table S3.** Weighted and unweighted Adonis tables for MaxFat, LoinThickness, Age, and RumpFat. **Table S4**. Phylum level correlations for taxa (December samples only) with March health outcomes in repeat deer. **Table S5**. Order level correlations for taxa (December samples only) with March health outcomes in repeat deer. **Table S6**. Family level correlations for taxa (December samples only) with March health outcomes in repeat deer. **Table S7**. Genus level correlations for taxa (December samples only) with March health outcomes in repeat deer. **Table S8**. ASV level correlations for taxa (December samples only) with March health outcomes in repeat deer. **Table S9**. Phylum level correlations for taxa (all samples) with the health measures of deer at time of sample collection. **Table S10**. Order level correlations for taxa (all samples) with the health measures of deer at time of sample collection. **Table S11**. Family level correlations for taxa (all samples) with the health measures of deer at time of sample collection. **Table S12**. Genus level correlations for taxa (all samples) with the health measures of deer at time of sample collection. **Table S13**. ASV level correlations for taxa (all samples) with the health measures of deer at time of sample collection.

**Additional file 2 Figure S1**. Alpha Rarefaction Curve. Rarefaction curve suggests optimal sequencing depth for rareified samples to be approximately 13,000 reads. 104 samples out of 108 are retained.

## Data Availability

Raw data are included as supplementary material or are available under accession numbers listed in the methods section.

## Abbreviations

rRNA: ribosomal ribonucleic acid
IFBF: Ingensta free body fat
PCoA: Principal coordinate analysis
ASV: Amplicon sequence variant
RFI: Residual feed intake
GFE: Gross feed intake
BYU: Brigham Young University
BCS: Body condition score
V4: Variable 4
FDR: False discovery rate
DF: Degrees of freedom
SS: Sum of squares
MS: Mean sum of squares
F: F-value
P: *p*-value
R_S_: Spearman’s rho
