# Supplementary material for: Spatiotemporal variation in the fecal microbiota of mule deer is associated with proximate and future measures of host health
Source: BMC Vet Res. 2021 Jul 29;17:258. doi: 10.1186/s12917-021-02972-0 (PMC8323208; doi:10.1186/s12917-021-02972-0)
Supplement: Supplementary file 2 — Additional file 2 Figure S1. Alpha Rarefaction Curve. Rarefaction curve suggests optimal sequencing depth for rareified samples to be approximately 13,000 reads. 104 samples out of 108 are retained. [file 12917_2021_2972_MOESM2_ESM.docx]

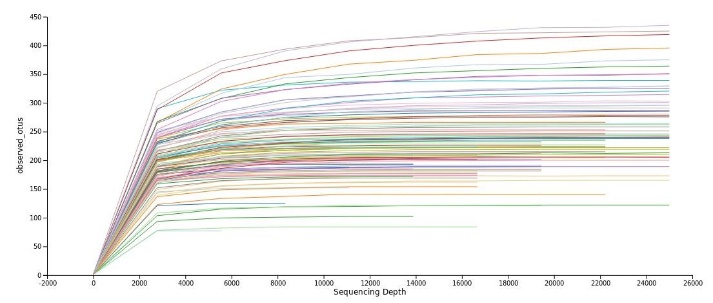


**Figure S1. Alpha Rarefaction Curve.** Rarefaction curve suggests optimal sequencing depth for rareified samples to be approximately 13,000 reads. 104 samples out of 108 are retained.
